# Evidence for Mechanistic Alterations of Ca^2+^ Homeostasis in Type 2 Diabetes Mellitus

**DOI:** 10.1155/EDR.2000.275

**Published:** 2000

**Authors:** Muthuswamy Balasubramanyam, Ramalingham A. Balaji, Balakrishnan Subashini, Viswanathan Mohan

**Affiliations:** 1 Center for Biotechnology Anna University Chennai 600025 India; 2 Madras Diabetes Research Foundation Gopalapuram Chennai 600086 India; 3 Madras Diabetes Research Foundation (MDRF) 35, Conran Smith Road Gopalapuram Chennai 600086 India

## Abstract

Altered cytosolic Ca^2+^ is implicated in the aetiology of many diseases including diabetes but there are few studies on the mechanism(s) of the altered Ca^2+^ regulation. Using human lymphocytes, we studied cytosolic calcium (Ca_i_) and various Ca^2+^ transport mechanisms in subjects with Type 2 diabetes mellitus and control subjects. Ca^2+^-specific fluorescent probes (Fura-2 and Fluo-3) were used to monitor the Ca^2+^ signals. Thapsigargin, a potent and specific inhibitor of the sarco(endo)plasmic reticulum Ca^2+^-ATPase (SERCA), was used to study Ca^2+^- store dependent Ca^2+^ fluxes. Significant (*P* < 0.05) elevation of basal Ca_i_ levels was observed in lymphocytes from diabetic subjects. Ca_i_ levels were positively correlated with fasting, plasma glucose and HbAlc. There was also a significant (*P* < 0.05) reduction in plasma membrane calcium (PMCA) ATPase activity in diabetic subjects compared to controls. Cells from Type 2 diabetics exhibited an increased Ca^2+^ influx (as measured both by Fluo-3 fliorescence and C45a assays) as a consequence of of thapsigargin-mediated Ca^2+^ store depletion. Upon addition of Mn^2+^ (a surrogate of Ca^2+^), the fura-2 fluorescence decayed in an exponential fashion and the rate and extent of this decline was steeper and greater in cells from type 2 diabetic patients. There was also a significant (*P* < 0.05) difference in the Na^+^/Ca^2+^ exchange activity in Type 2 diabetic patients, both under resting conditions and after challenging the cells with thapsigargin, when the internal store Ca^2+^ sequestration was circumvented. Pharmacological activation of protein kinase C (PKC) in cells from patients resulted in only partial inhibition of Ca^2+^ entry. We conclude that cellular Ca^2+^ accumulation in cells from Type 2 diabetes results from (a) reduction in PMCA ATPase activity, (b) modulation of Na^+^/Ca^2+^ exchange and (3) increased Ca^2+^ influx across the plasma membrane.


*Int. Jnl. Experimental Diab. Res.*, Vol. 1, pp. 275–287
Reprints available directly from the publisher
Photocopying permitted by license only

© 2001 OPA (Overseas Publishers Association) N.V.
Published by license under
the Harwood Academic Publishers imprint,
part of The Gordon and Breach Publishing Group.
Printed in Malaysia.

### Evidence for Mechanistic Alterations of $Ca^{2+}$ Homeostasis in Type 2 Diabetes Mellitus

MUTHUSWAMY BALASUBRAMANYAM[a,*], RAMALINGHAM A. BALAJI[a], BALAKRISHNAN SUBASHINI[a] and VISWANATHAN MOHAN[b]

[a]*Center for Biotechnology, Anna University, Chennai-600025, India;*
[b]*Madras Diabetes Research Foundation, Gopalapuram, Chennai-600086, India*

*(Received 8 November 1999; In final form 17 June 2000)*

**Altered cytosolic $Ca^{2+}$ is implicated in the aetiology of many diseases including diabetes but there are few studies on the mechanism(s) of the altered $Ca^{2+}$ regulation. Using human lymphocytes, we studied cytosolic calcium ($Ca_i$) and various $Ca^{2+}$ transport mechanisms in subjects with Type 2 diabetes mellitus and control subjects. $Ca^{2+}$-specific fluorescent probes (Fura-2 and Fluo-3) were used to monitor the $Ca^{2+}$ signals. Thapsigargin, a potent and specific inhibitor of the sarco(endo)plasmic reticulum $Ca^{2+}$-ATPase (SERCA), was used to study $Ca^{2+}$-store dependent $Ca^{2+}$ fluxes. Significant ($P<0.05$) elevation of basal $Ca_i$ levels was observed in lymphocytes from diabetic subjects. $Ca_i$ levels were positively correlated with fasting plasma glucose and HbA1c. There was also a significant ($P<0.05$) reduction in plasma membrane calcium (PMCA) ATPase activity in diabetic subjects compared to controls. Cells from Type 2 diabetics exhibited an increased $Ca^{2+}$ influx (as measured both by Fluo-3 fluorescence and $^{45}Ca$ assays) as a consequence of thapsigargin-mediated $Ca^{2+}$ store depletion. Upon addition of $Mn^{2+}$ (a surrogate of $Ca^{2+}$), the fura-2 fluorescence decayed in an exponential fashion and the rate and extent of this decline was steeper and greater in cells from type 2 diabetic patients. There was also a significant ($P<0.05$) difference in the $Na^+/Ca^{2+}$ exchange activity in Type 2 diabetic patients, both under resting conditions and after challenging the cells with thapsigargin, when the internal store $Ca^{2+}$ sequestration was circumvented. Pharmacological activation of protein kinase C (PKC) in cells from patients resulted in only partial inhibition of $Ca^{2+}$ entry. We conclude that cellular $Ca^{2+}$ accumulation in cells from Type 2 diabetes results from (a) reduction in PMCA ATPase activity, (b) modulation of $Na^+/Ca^{2+}$ exchange and (3) increased $Ca^{2+}$ influx across the plasma membrane.**

*Keywords*: Type 2 diabetes; Calcium; PMCA; SERCA; Na/Ca exchange

### INTRODUCTION

Recent reports indicate that Asian Indians as a race have a high prevalence of diabetes.[1] Indeed, India has the largest number of diabetic patients in the world and these numbers are expected to further increase in the next few decades.[2] Type 2 diabetes mellitus (non-insulin-dependent diabetes mellitus, NIDDM) is the most common form representing over 85% of all

*Address for correspondence: Madras Diabetes Research Foundation (MDRF), 35, Conran Smith Road, Gopalapuram, Chennai 600086, India. Fax: 91-44-8258935, e-mail: drmohan@giasmd01.vsnl.net.in diabetes cases,[3] and is commonly accompanied by elevated blood pressure. Type 2 diabetes is characterised by relative insulin deficiency and insulin resistance, but the cellular and molecular mechanism(s) of these defects remain unclear.

There is increasing evidence that $Ca^{2+}$ plays an important regulatory role in the cascade of insulin-generated signals [4–6] and $\beta$-cell function.[7] Elevated or sustained levels of cytosolic calcium ($Ca_i$) has been shown to diminish cellular sensitivity to insulin and might participate in the pathogenesis of insulin resistance in Type 2 diabetes and in various diabetes-related metabolic derangements.[8–11] Altered cellular $Ca^{2+}$ homeostasis could also well be an important mechanism for abnormal glucose metabolism and elevated blood pressure in Type 2 diabetes patients. Although the potential importance of the observed increases in cellular $Ca^{2+}$ is clear, the mechanism(s) responsible for this elevation of $Ca_i$ has not been identified. Therefore, mechanistic $Ca^{2+}$ turnover studies could provide a basis for better understanding at the cellular and molecular level of the long recognised clinical linkage between cardiovascular and metabolic syndromes. These intermediate phenotypes are also essential tools to fill the gap between gene polymorphism and complex diseases.

It is conceivable that the signal transduction defects of the $Ca^{2+}$ messenger system in the blood cells would reflect similar disturbances in target tissues affected in diabetes and diabetes-associated complications. Additionally, measurements of intracellular cations using circulating blood cells have been shown to be highly reproducible and the phenotypic characteristics persist even in cell culture models.[12] Lymphocytes were used in this study because they are readily accessible, their cytosolic calcium regulation is well understood[13, 14] and more importantly they could potentially provide genomic DNA for studying the underlying genetic mechanisms of diabetic complications.

### MATERIALS AND METHODS

### Materials

'Lymphoprep' was obtained from GIBCO Life Technologies (Gaithersburg, MD, USA). Thapsigargin, ionomycin, Fura-2 AM and Fluo-3 AM, phorbol 12-myristate 13-acetate (PMA), ouabain and other coupled-enzyme chemicals were from Sigma Chemicals (St Louis, MO, USA). $^{45}Ca^{2+}$ was purchased from Amersham (Amersham-Pharmacia Biotech, India). All other buffer chemicals were of analytical grade.

### Subjects and Blood Chemistry Assays

This study was performed in 30 Type 2 diabetes patients and 30 age and weight matched control subjects, recruited from the M.V.Diabetes Specialities Centre, Chennai, formerly Madras in Southern India. Diagnosis of Type 2 diabetes was based on the criteria of the World Health Organization study group on diabetes.[15] Informed consent was obtained from all study subjects. Blood samples and blood pressure measurements were taken between 0700 and 0900 h after an overnight fast. Blood (20 ml) drawn from each subject in acid citrate dextrose buffer was used for lymphocyte $Ca^{2+}$ studies. An additional 10 ml blood treated with EGTA was used for measurement of blood chemistry parameters. The study protocol was approved by the ethical committee of Centre for Biotechnology, Anna University. All biochemical studies were done on Corning Express plus Auto Analyzer (Corning, USA). Fasting plasma glucose (glucose oxidase method) was estimated using kits from Boehringer Mannheim, Germany. Glycosylated haemoglobin (HbAlc) were estimated by high-pressure liquid chromatography (HPLC) method using the Variant machine (Bio Rad, Hercules, USA). Lipid profile and serum creatinine were assayed using commercial kits (Boehringer Mannheim, Germany).

### Isolation of Lymphocytes from Whole Blood

Lymphocytes were isolated by the method of density gradient centrifugation[16] with modifications described earlier.[13] Blood was diluted with an equal volume of HEPES buffered solution (HBS) and layered onto lymphoprep (2:1 vol/vol). After density gradient centrifugation (45 min at 1700 rpm), lymphocytes were collected from the interface, diluted 1:1 with HBS. The lymphocyte pellet that resulted from a second centrifugation at 1600 rpm for 10 min was resuspended in Hepes buffer.

### Membrane Preparation and Coupled-enzyme Assay

The cells resuspended in a lysis medium containing 62.5 mmol/l Tris-HCl, pH 6.8, 1% SDS, 5 mole/l Urea, 10% glycerol with appropriate protease inhibitors, were disrupted by low ultrasonication (300 cycles) for 3 min. The lysed cells were centrifuged at 500g for 15 minutes (4°C) and the supernatant was further spun at 14,000g for 45 min (4°C). The final membrane pellet was resuspended at a concentration of 5 mg/ml.

The rate of $Ca^{2+}$ dependent ATP hydrolysis was determined by a coupled-enzyme system.[17] The composition of the final mixture for the assay medium was 120 mmol/l KCl, 30 mmol/l HEPES, 2.5 mmol/l $MgCl_2$, 1 IU/ml pyruvate kinase, 1 IU/ml lactate dehyrogenase, 120 μg/ml phosphoenol pyruvate, 150 μg/ml of NADH, 10–20 μg membrane protein, 5 μM ionomycin, and 1mmol/l ATP (pH 7.0 at 37°C). The membranes were preincubated with or without thapsigargin for 5 min prior to the start of the reaction and introduced into the ATP containing assay medium. The oxidation of NADH was monitored at 340 nm in a Hitachi Spectrophotometer (model U-3210, Japan), which directly indicated the hydrolysis of ATP by the $Ca^{2+}$ ATPase. Free $Ca^{2+}$ in the medium was adjusted by EGTA and $CaCl_2$ additions, using a computer program. $Ca^{2+}$ dependent hydrolysis of ATP was determined by subtracting the rate of activity in the presence of EGTA from the rate in the presence of $Ca^{2+}$.

### $^{45}Ca^{2+}$ Uptake in Intact Lymphocytes

Lymphocytes were preincubated with (for $Na^+/Ca^{2+}$ exchange assay) or without (for $Ca^{2+}$ entry assay) 0.1 mmol/l ouabain in $Na^+$ containing medium for 30 min. To initiate $Ca^{2+}$ uptake, aliquots of cells ($1 \times 10^8$ cells/ml) were diluted into $Na^+$ containing medium or $Na^+$ free medium (NaCl is iso-osmotically replaced by NMDG) containing 10 μCi/ml $^{45}CaCl_2$. Ouabain pretreatment was used to increase cytosolic $Na^+$ through inhibition of the $Na^+/K^+$ ATPase.[14] In experiments where the cells were treated with thapsigargin (Tg), 100 nmol/l Tg was included in the assay medium. After one minute incubation at 37°C, $Ca^{2+}$ uptake was stopped by addition of 5 ml of ice-cold stopping buffer (40 mmol/l Hepes, 100 mmol/l $MgCl_2$, 5 mmol/l $LaCl_3$). Extracellular radioactivity was removed by rapid filtration of cells on 0.45 μ filters with two additional washes of stopping buffer. Each data point represents the mean of '*n*' experiments derived from triplicate or quadruplicate measurements. Background counts (medium without cells) were substracted from all experimental time points.

### Fluorescent Dye Loading $Ca_i$ Monitoring

Lymphocytes were incubated with 5 μmol/l Fura-2 or 5 μmol/l Fluo-3 for a minimum of 30 min at 37°C and delivered as 100 μl ($0.5 \times 10^6$ cells/ml) aliquots. Prior to each experiment, cells were centrifuged for 5–10 sec at room temperature, resuspended in 100 μl of HBS, and injected into cuvettes containing 3 ml of assay buffer. $Ca_i$ monitoring using Fluo-3 was monitored in a Hitachi spectrofluorimeter (model F-3010, Japan) by excitation wavelength set at

490 nm and emission wavelength at 525 nm. Calibration was achieved by exposing newly resuspended lymphocytes to digitonin and EGTA to get $F_{max}$ and $F_{min}$ values.[13] From the fluorescence values ($F$), the $Ca_i$ was calculated according to the method of Grynkiewicz *et al.* [18] utilizing the dissociation constant of the dye (Kd = 400 nM), $F_{max}$ and $F_{min}$ values. Autofluorescence of the unloaded cells was substracted from the fluorescence values.

### $Mn^{2+}$ Quench Experiments

$Mn^{2+}$ has been used as a $Ca^{2+}$ surrogate to study $Ca^{2+}$ entry mechanisms [13]. We thus monitored the rate of quenching of intracellular fura-2 by $Mn^{2+}$ as a measure of $Ca^{2+}$ entry across the plasma membrane. This analysis was performed by following the decay of fura 2 fluorescence at excitation wavelength 360 nm in lymphocytes suspended in $Ca^{2+}$-HBS and incubated with 0.5 mmol/l $MnCl_2$.

### Data Analysis

Statistical analysis was performed with Student's $t$ test, one-way analysis of variance and correlation analyses. $P < 0.05$ was considered to be statistically significant. Data in the figures are expressed as Means ± SE.

### RESULTS

Clinical characteristics of the control and Type 2 diabetes subjects are summarised in Table I. Diabetic patients had significantly ($P < 0.05$) higher body mass index, fasting plasma glucose, cholesterol, HbA1c and systolic blood pressure than controls. $Ca_i$ was positively correlated with fasting plasma glucose ($r$ = 0.4752, $p$ = 0.05) and Hb1Ac ($r$ = 0.5617, $p$ = 0.05).

The basal $Ca_i$ measurement performed using Fluo-3 in lymphocytes is shown in Figure 1. The resting $Ca_i$ in lymphocytes from Type 2 diabetes (57.4 nmol/l) was signficantly higher than in cells from control (43.7 nmol/l) subjects. Because changes in $Ca_i$ result from a number of biochemical processes, we looked into specific $Ca^{2+}$ transport pathways that govern $Ca^{2+}$ homeostasis. Figure 2 shows the $Ca^{2+}$ ATPase activities (µmole/mg protein/min) in lymphocytes from control and Type 2 diabetic individuals respectively. Thapsigargin (Tg), a novel inhibitor of the SERCA-ATPase(s),[19, 20] was used to determine the relative contribution of different $Ca^{2+}$ ATPase activities. Tg-resistant component was considered as plasma membrane calcium (PMCA) ATPase activity and sarco(endo)plasmic calcium (SERCA) ATPase activity as Tg-sensitive component. In control cells, total activity (0.179 ± 0.013 µmole/mg protein/min) comprises 62% of Tg-resistant (0.110 ± 0.008) and 38% of Tg-sensitive (0.068 ± 0.01) activities. In contrast, the total $Ca^{2+}$ ATPase activity (0.147 ± 0.013 µmole/mg protein/min) in cells from Type 2 diabetes was represented by an equal share of Tg-resistant (0.073 ± 0.007) and Tg-sensitive (0.073 ± 0.007) components. While there was no significant difference in SERCA ATPase activities in membranes from control and patients, lympocyte

TABLE I Clinical Characteristics of study subjects

| Parameters | Control ($n$ = 30) | Type 2 diabetes ($n$ = 30) |
|---|---|---|
| Age (years) | 50.6 ± 11 | 54.4 ± 11 |
| BMI (kg/m$^2$) | 23.6 ± 4.6 | 31.9 ± 9.1* |
| Systolic blood pressure (mm Hg) | 127 ± 17 | 136 ± 13* |
| Diastolic blood pressure (mm Hg) | 79 ± 10 | 81 ± 11 |
| Fasting plasma glucose (mg/dl) | 81 ± 14 | 163 ± 48* |
| HbAlc (%) | 6.0 ± 0.4 | 8.1 ± 1.7* |
| Cholesterol (mg/dl) | 173 ± 34 | 193 ± 35* |
| Triglyceride (mg/dl ) | 111 ± 39 | 132 ± 56 |
| HDL (mg/dl) | 41 ± 10 | 38 ± 11 |
| LDL (mg/dl) | 116 ± 32 | 128 ± 39 |
| Creatinine (mg/dl) | 0.8 ± 0.2 | 0.9 ± 0.2 |

Values are Means ± SD.

* $P < 0.05$.

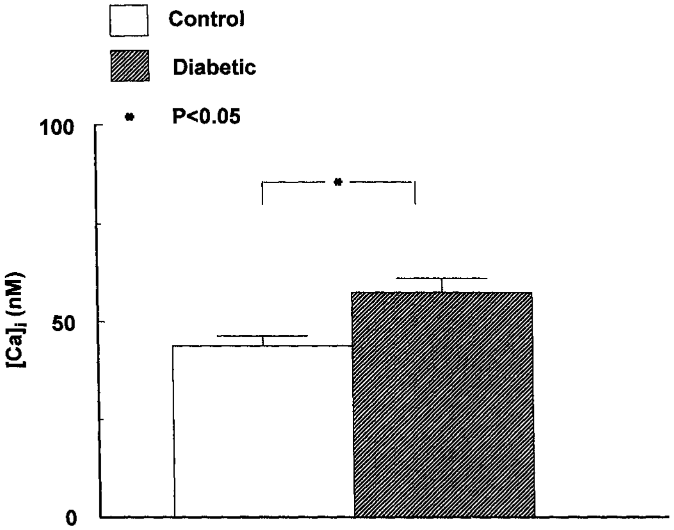

FIGURE 1 Basal $Ca_i$ levels in lymphocytes from control ($n = 12$) and Type 2 diabetes ($n = 12$) subjects.

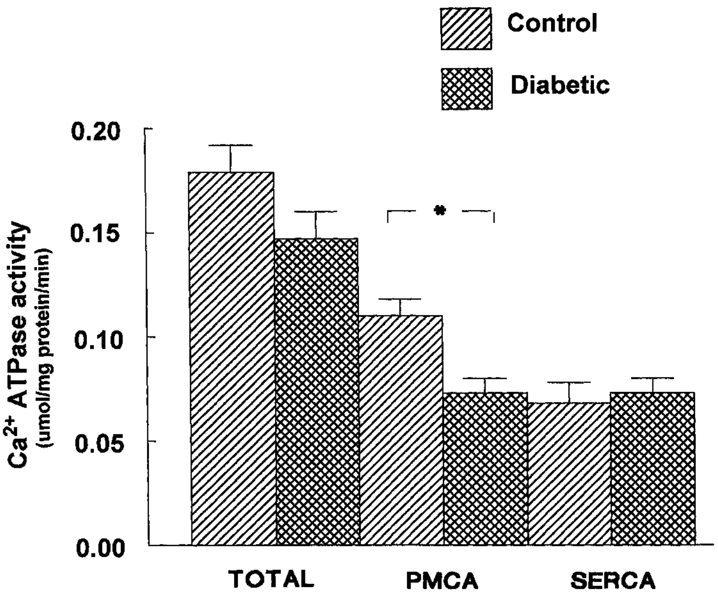

FIGURE 2 Mean $Ca^{2+}$-ATPase activities in control and Type 2 diabetes subjects. Thapsigargin (Tg) was used to determine Tg-sensitive (SERCA) and Tg-resistant (PMCA) ATPase activities, as detailed in the Methods. Tg-resistant (PMCA) ATPase activities in cells from control and Type 2 diabetes subjects differ significantly ($P < 0.05$).

membranes from Type 2 diabetic patients exhibit lower levels ($P < 0.05$) of PMCA ATPase activity (Fig. 2). Because inhibition of either the PMCA or SERCA ATPases could lead to an increase in cytosolic $Ca^{2+}$, the data suggests that there is a defect in PMCA ATPase mediated $Ca^{2+}$ extrusion mechanism.

$Na^{+}$ dependent $^{45}Ca$ uptake assay was used to determine the $Na^{+}/Ca^{2+}$ exchange activity, a maneuver that involves raising of the cytosolic

$Na^+$ concentration and/or lowering of the external $Na^+$ concentration. The protocol for measuring $Na^+$ dependent $Ca^{2+}$ uptake assay is illustrated in Figure 3. Ouabain pretreatment was used to inhibit the Na–K-ATPase, increase $Na_i$ and decrease the inwardly directed Na gradient that inhibits Ca entry by 'forward mode' $Na^+/Ca^{2+}$ exchange. A greater accumulation of $Ca^{2+}$ uptake seen in $Na^+$-free conditions is the evidence for $Na^+/Ca^{2+}$ exchange activity (Fig. 3). Per cent $Na^+/Ca^{2+}$ exchange was calculated by substracting the $^{45}Ca^{2+}$ uptake in $Na^+$-containing medium from that obtained in $Na^+$ free conditions (inset). The composite results of % $Na^+/Ca^{2+}$ exchange derived from a number of similar experiments with and without Tg and in control and Type 2 diabetes subjects are summarised in Figure 4. In control cells, the reversal of $Na^+$ gradient resulted in a net $Ca^{2+}$ uptake mediated by $Na^+/Ca^{2+}$ exchange (70.4%) that increased to 92.2% in the presence of Tg. The same maneuver measured only 36% $Na^+/Ca^{2+}$ exchange activity in cells from Type 2 diabetes, but this significantly ($P < 0.05$) increased to 137.2% in the presence of Tg. These results suggest that $Na^+/Ca^{2+}$ exchange activity is depressed in Type 2 diabetes patients under resting, unstimulated conditions. However, augmented $Na^+/Ca^{2+}$ exchange was noticed in cells from both control and Type 2 diabetic subjects after treatment with Tg. Indeed, cells from Type 2 diabetes incubated with Tg, showed a 3 fold increase in $Na^+/Ca^{2+}$ exchange activity when compared to resting cells ($Na^+/Ca^{2+}$ exchange activity recorded as an increase from 36% to 137%). This means that the internal stores undoubtedly play a crucial role in buffering increases in net $Ca^{2+}$ gain occurred through

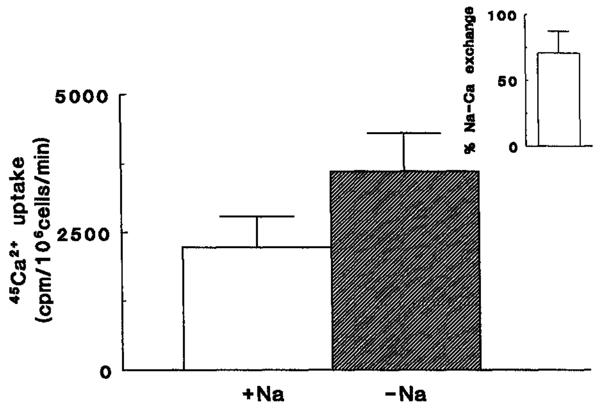

FIGURE 3 Protocol for measuring $Na^+$-dependent $Ca^{2+}$ influx. Lymphocytes were preincubated for 30 min at 37°C in $Na^+$ medium with or without 0.1mmol/l ouabain and resuspended in $Na^+$-or $Na^+$-free medium containing $^{45}Ca^{2+}$. $Ca^{2+}$ uptake was measured after 60 sec; each experiment was performed in quadruplicate and averaged. Data represent mean values of 6 individual experiments. % $Na^+$–$Ca^{2+}$ exchange (inset) was calculated by subtracting the values of $Ca^{2+}$ uptake in $Na^+$ medium from $Na^+$-free medium.

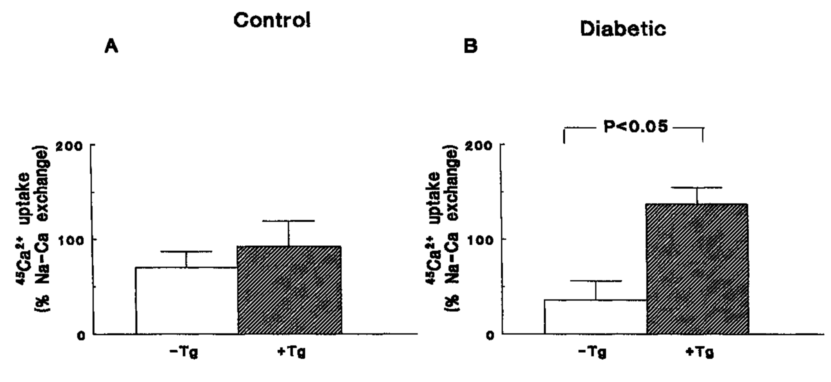

FIGURE 4 Percentage $Na^+/Ca^{2+}$ exchange in lymphocytes from control (A) and Type 2 diabetes (B) subjects in the presence and absence of thapsigargin (Tg). The methodology for these experiments is exactly the same as in Figure 3 and % $Na^+/Ca^{2+}$ exchange values (each representing mean of 6 separate cell preparations) are shown combined.

$Na^+/Ca^{2+}$ exchange. In the presence of Tg, the internal store $Ca^{2+}$ buffering was circumvented.

Since the alterations in resting $Ca_i$ could be a manifestation of changes occurring through many mechanisms, we next looked into the $Ca^{2+}$ entry ($Ca^{2+}$ uptake) processes across the plasma membrane. $^{45}Ca^{2+}$ uptake (one min) measurements in the presence and absence of Tg, in cells from control and Type 2 diabetic patients respectively, are depicted in Figure 5. Tg-mediated $Ca^{2+}$ uptake over the resting $Ca^{2+}$ uptake was significantly ($P < 0.05$) higher in both control and Type 2 diabetic patient cells. However, Tg-stimulated $Ca^{2+}$ entry was significantly higher (47.8%) in cells from Type 2 diabetics compared to control lymphocytes (23.2%) (insets). Measurement of $Ca_i$ in lymphocytes from control and Type 2 diabetes subjects also indicates a differential profile of store-operated $Ca^{2+}$ influx (Figs. 6A and B). Cells from Type 2 diabetes exhibited an increased initial rate of Tg-evoked $Ca^{2+}$ influx when compared to the similar profile in control cells. As depicted in Figure 6C, mean $Ca_i$ levels at 60 sec after the addition of extracellular Ca (comparable to the one min $^{45}Ca^{2+}$ uptake) were significantly ($P < 0.05$) different in cells from control (162 nmol/l) and patients (235 nmol/l). Figure 7 illustrates the role of Tg-evoked $Mn^{2+}$ influx as another experimental maneuver for studying store-operated $Ca^{2+}$ influx. Upon addition of $Mn^{2+}$, the fura-2 fluorescence decayed in an exponential fashion and the rate and the extent of this decline was steeper and greater in cells from Type 2 diabetes. The initial rate of $Mn^{2+}$ entry is a parameter that reflects the opening state of the $Ca^{2+}$ influx pathway.

Phorbol esters, such as PMA activate PKC and alter multiple cellular responses. We incubated lymphocytes with PMA, and monitored the $Ca_i$ responses after treatment with Tg (Figs. 6A and B). Though in general, PKC activation resulted in inhibition of $Ca^{2+}$ influx, the extent of this inhibition in cells from Type 2 diabetic subjects (38%) was substantially lower than control (68%) subjects (Fig. 6D). This was also supported by $Mn^{2+}$ entry experiments (Figs. 7A and B).

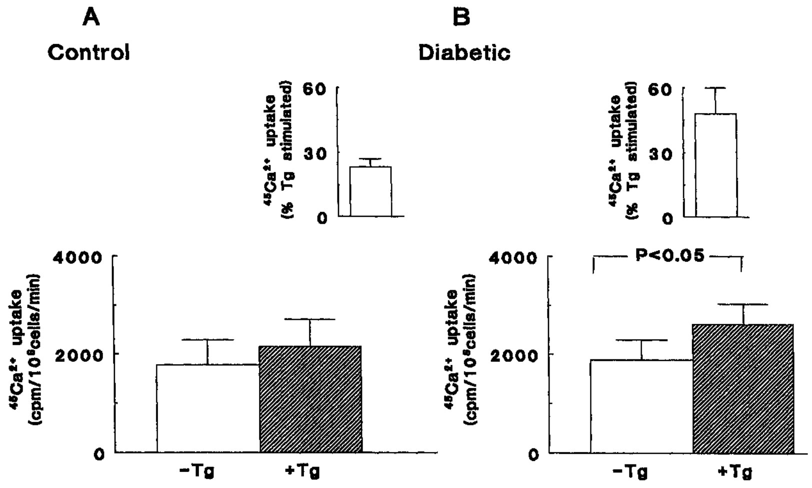

FIGURE 5 $^{45}Ca^{2+}$ uptake in lymphocytes from control (A) and Type 2 diabetes (B) subjects. Lymphocytes were preincubated for 30 min at 37°C and resuspended in $Na^+$ medium containing $^{45}Ca^{2+}$. $Ca^{2+}$ uptake was measured after 60 sec with or without Tg in the medium. Each experiment was performed in quadruplicate and averaged. Data represent mean values of 6 individual experiments. % Tg-stimulated $^{45}Ca^{2+}$ uptake was calculated by subtracting the values of $Ca^{2+}$ uptake in the presence of Tg from that in the absence of Tg (Insets).

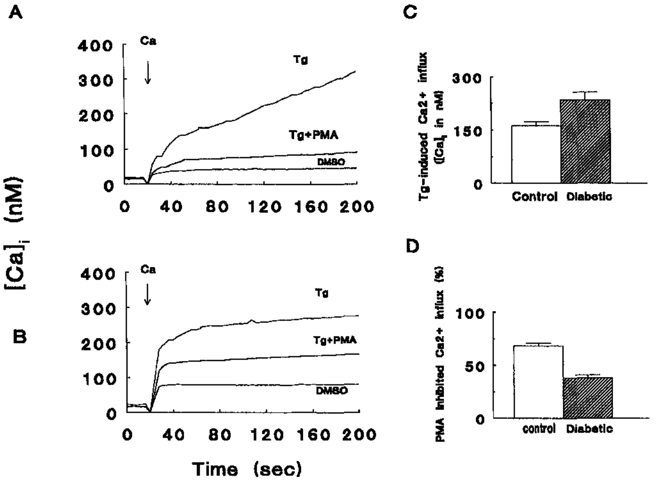

FIGURE 6 Tg-induced $Ca^{2+}$ store-operated $Ca^{2+}$ entry and its inhibition by PKC activation in control (A) and Type 2 diabetes (B). Lymphocytes were subjected to 100 nmol/l Tg in $Ca^{2+}$-free HBS for 2 min and pretreated with and without 100 nmol/l PMA. $Ca^{2+}$ was then introduced in to the cuvette (arrow) to raise $[Ca^{2+}]_e$ to 1 mmol/l and $Ca_i$ profile was subsequently followed for 3 min. Representative traces of 6 separate experiments. C: Mean values ($n = 6$) of Tg-induced $Ca^{2+}$ influx in control and Type 2 diabetes subjects as calculated from the $Ca_i$ traces in the figure(s) after 60 sec to the addition of $CaCl_2$. D: Mean percentage ($n = 6$) of PMA inhibited Tg-induced $Ca^{2+}$ influx in control and Type 2 diabetes subjects.

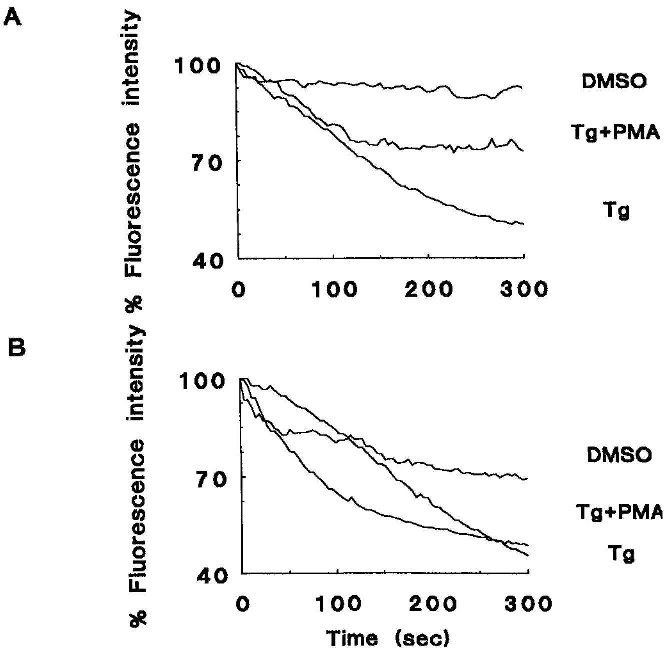

FIGURE 7 $Mn^{2+}$ entry ($Ca^{2+}$ influx) in lymphocytes from control (A) and Type 2 diabetes (B) subjects. Lymphocytes were resuspended in $Ca^{2+}$-HBS and followed at 360 nm excitation wavelength. Fluorescence reading immediately before addition of 0.5 mmol/l $MnCl_2$ (0 sec) was set at 100% intensity. $Mn^{2+}$ quenching of cellular fura 2 was expressed as percentage of this value. In experiments where PMA was used, lymphocytes were pre-incubated with the phorbol ester for 2 min and the fluorescence monitoring was started with the addition of Tg and 0.5 mmol/l $MnCl_2$. Representative traces of 6 separate experiments.

### DISCUSSION

Several studies have demonstrated elevated $Ca_i$ in cells from diabetic and/or hypertensive individuals[10, 21–28] and a reduction in $Ca_i$ associated with a combination drug therapy.[29] The fine tuning of the cell $Ca^{2+}$ is primarily performed by two high $Ca^{2+}$ affinity pumps under the control of SERCA and PMCA ATPases. A calmodulin-stimulated ATP-dependent $Ca^{2+}$ uptake and a corresponding PMCA ATPase have been described in lymphocytes.[30] Balasubramanyam *et al.* [13] have also demonstrated fluorimetrically a well defined $Ca^{2+}$ extrusion process in freshly isolated human peripheral blood lymphocytes, which is mediated by Ca pump of the plasma membrane. The present study shows that there is an impairment in $Ca^{2+}$ turnover in Type 2 diabetic subjects with a significant reduction of PMCA ATPase activity. In diabetic patients as well as in experimental diabetes mellitus, there are conflicting results on cellular $Ca^{2+}$ ATPase with the reports of decreased [31–37] and increased [22, 38–39] activities. Our results support the work of Spieker *et al.* [36] who demonstrated decreased $Ca^{2+}$-ATPase activity in erythrocytes from both Type 1 and Type 2 diabetic patients. Similar inhibition of PMCA activity related to higher $Ca_i$ levels in platelets from hypertensive individuals has also been reported.[40, 41] The mechanism(s) of inhibition of PMCA ATPase in Type 2 diabetes might be attributed to increased protein phosphatase activity, modulation of protein kinase A or C, or both.[42] While alterations in lipid composition in patients (changes in both membrane fluidity and acidic phospholipid content) have been shown to affect the plasma membrane $Ca^{2+}$ pump, high glucose in uncontrolled diabetes would also probably leads to glycosylation of the PMCA pump and its inhibition.[43]

Apart from PMCA ATPase, the other important transport process that can mediate net $Ca^{2+}$ extrusion across the plasma membrane is $Na^+/Ca^{2+}$ exchange. Our findings also suggest a possible role for $Na^+/Ca^{2+}$ exchange in $Ca^{2+}$ regulation in lymphocytes and its modulation in diabetes states. A primary increase in $Na^+$ permeability,[44] an increase in intracellular $Na^+$ caused by a circulating inhibitor of the sodium pump [45] or an increase in $Na^+$ due to enhanced activity of the $Na^+/H^+$ exchanger could all promote $Na^+$-dependent $Ca^{2+}$ influx *via* $Na^+/Ca^{2+}$ exchange leading to the observed increase in $Ca_i$. Pharmacologically, ouabain is routinely used in $Na^+$-dependent $Ca^{2+}$ uptake studies. Lymphocytes exposed to 0.1 mmol/l ouabain in $Na^+$ medium for 30 min demonstrated elevated (~40 mM) levels of cytosolic $[Na^+]$, as measured using $Na^+$-specific SBFI fluorescent indicator.[14] Using this and other maneuvers, Gardner and Balasubramanyam[46] have demonstrated the existence of $Na^+$ dependent $Ca^{2+}$ entry pathway in peripheral *T*-lymphocytes and other cell lines. Our methodology to measure $Na^+/Ca^{2+}$ exchange activity in cells from Type 2 diabetes provides evidence that $Na^+/Ca^{2+}$ exchange activity is low in resting conditions and that depletion of intracellular $Ca^{2+}$ stores by Tg resulted in a 3 fold increase in its activity. It is important to note that $Na^+/Ca^{2+}$ exchange is a completely reversible electrogenic transport reaction, and the operation of the carrier is controlled by the magnitude and polarity of transmembrane electrical potentials and ionic gradients. Hence, depending on prevailing physiological conditions, the transport could provide either a net cellular efflux or influx of $Ca^{2+}$. In the light of findings that the $Na^+/K^+$ ATPase may be inhibited by endogenous ouabain in some hypertensive and/or diabetic subjects,[45, 47] increased cytosolic $Na^+$ may indeed have a causal role in the regulation of cytosolic $Ca^{2+}$ and/or $Ca^{2+}$ content of intracellular stores. The present study indicates that a reduction in normal extrusion mode of $Na^+/Ca^{2+}$ exchange activity (or the reverse-mode switch over) could be one of the mechanisms that play a role in diabetes-mediated accumulation of cellular $Ca^{2+}$.

Studies in lymphocytes and pancreatic $\beta$-cells have shown that the depletion of intracellular $Ca^{2+}$ stores,[13, 48] leads to activation of $Ca^{2+}$ influx occurring through plasma membrane-specific $Ca^{2+}$ channels referred to as "store-operated channels". Our measurements of $Ca^{2+}$ uptake/influx in lymphocytes from control and Type 2 diabetes subjects indicate an augmented store-operated $Ca^{2+}$ influx in cells from diabetics. The molecular nature of these store-operated ion channels have not been elucidated. Nonetheless, the Drosophila transient receptor potential (trp) gene has been suggested to encode a store-operated ion channel.[49] Recently a number of vertebrate homologues of 'trp' have been cloned and expression of these proteins has been demonstrated in a variety of tissues including vascular endothelial cells and pancreatic $\beta$-cells.[50–52] Trp-related ion conductance is likely to serve hormonal control of cell membrane potential and $Ca^{2+}$ homeostasis, and may therefore paly a significant role in physiology and pathophysiology. As suggested by Fekete *et al.*[53] the $[Ca^{2+}]_i$ response to store-depletion-activated $Ca^{2+}$ influx by agonists may serve as an intermediate phenotype for genetic linkage studies in hypertension and/or diabetes.

Higher Tg-stimulated $Ca^{2+}$ uptake/influx in cells from Type 2 diabetes indicate that there may be an impairment in mechanism(s) that regulate $Ca^{2+}$ influx. The physiological signal for $Ca^{2+}$ entry still remains a mystery. A role for kinases and phosphatases has been implicated.[54] Protein kinase C (PKC) is known to regulate $Ca^{2+}$ homeostasis through negative feedback mechanisms.[55] It is interesting to note that activation of feedback mechanisms such as PKC only partially respond to increased $Ca_i$ signals in cells from Type 2 diabetes. Infact, hyperglycemia has been reported to increase diacylglycerol and activate PKC activity in many tissues and is thus linked to the vascular complications.[56] It is presently not clear whether chronic PKC activation in Type 2 diabetics could down-regulate PKC and make the cells unresponsive to PKC-mediated $Ca_i$ regulation.

The positive correlation of $Ca_i$ with the plasma glucose and HbA1c values support the earlier views that both hyperglycemia and insulin deficiency affect $Ca_i$ regulation.[57–59] HbA1c levels, indicating the degree of metbolic control over a longer period of time, may exert a lasting effect on cellular $Ca_i$ and/or other intermediary mechanisms. Interestingly, not only can higher $Ca_i$ levels contribute to insulin resistance, but diabetes can also lead to significant alterations in cellular $Ca^{2+}$ handling.

The origin of the cellular $Ca^{2+}$ shifts in diabetes and/or hypertension is not clear. Genetic defects in cell membrane $Ca^{2+}$ transport or transport of $Ca^{2+}$ across the intracellular membrane systems may contribute to alterations in cellular $Ca^{2+}$ homeostsis. There is general agreement that genetic factors could be the primary determinants of impaired insulin secretion and action. Altered cellular and sub-cellular $Ca^{2+}$ homeostasis can cause abnormal insulin secretion, increased vascular resistance, and altered response of vascular smooth muscle cells to $Ca^{2+}$ mobilising vasoactive hormones.[60, 61] The vascular changes accompanied by an appreciable rise in systemic blood pressure may in turn, cause hypertension. Finally, as suggested by Wehling and Theisen,[62] hypertension and insulin resistance may have further deleterious effects on membrane phospholipid content and cellular $Ca^{2+}$ homeostasis, creating a vicious cycle.

This work has several implications for future studies. This is the first report of $Ca^{2+}$ turnover studies in Indian Type 2 diabetes patients. We have identified defects in $Ca^{2+}$ transport activities, *viz.*, ATPase(s), $Na^+/Ca^{2+}$ exchanger and store-operated $Ca^{2+}$ channels and these can be studied further using both proteomic and genomic approaches and lymphoblasts would be an ideal cell culture model for such studies. The fact that ion transport abnormalities are persistent in *in vitro* culture [63, 64] indicates that these phenotypic expressions may be genetically determined. More importantly, a recent study has already reported sequence variants of the SERCA 3 gene in white Caucasians with Type 2 diabetes [65] which needs to be studied in different ethnic diabetic populations. Until a true genetic marker is identified, cellular elements may serve as intermediate phenotypes for screening high-risk diabetic patients and as targets for the development of new therapeutic compounds. Continued research efforts on molecular and genetic studies of families of diabetic patients are warranted to identify the initial defect and its genetic component of altered $Ca^{2+}$ homeostasis.

### Acknowledgements

This work was supported by grants from the Third World Academy of Sciences (TWAS, Italy) and the Council of Scientific and Industrial Research (CSIR, India). The authors thank Prof. Kunthala Jayaraman and Prof. P. Kaliraj, Center for Biotechnology, Anna University, Chennai for their assistance.

### References

[1] Ramachandran, A., Snehalatha, C., Latha, E., Vijay, V. and Viswanathan, M. (1997). Rising prevalence of NIDDM in an urban population in India, *Diabetologia*, **40**, 232–237.

[2] King, H., Auberti, R. E. and Herman, W. H. (1998). Global burden of diabetes 1995–2025: Prevalence, numerical estimates and projections, *Diabetes Care*, **21**, 1414–1431.

[3] World Health Organisation (WHO). (1994). Prevention of diabetes mellitus. Report of a WHO study group, Geneva, *World Health Org., Techl. Rep. Ser.*, **844**.

[4] Draznin, B., (1988). Intracellular calcium, insulin secretion, and action, *Am. J. Med.*, **85**, 44–58.

[5] Bertuzzi, F., Zacchetti, D., Berra, C., Socci, C., Pozza, G., Pontiroli, A. E. and Grohovaz, F., (1996). Intercellular $Ca^{2+}$ waves sustain coordinated insulin secretion in pig islets of Langerhans, *FEBS Lett.*, **379**, 21–25.

[6] Bergsten, P., Lin, J. and Westerlund, J. (1998). Pulsatile insulin release: role of cytoplasmic $Ca^{2+}$ oscillations, *Diab. Metab.*, **24**, 41–45.

[7] Berggren, P. O. and Larsson, O. (1994). $Ca^{2+}$ and pancreatic b-cell function, *Biochem. Soc. Trans.*, **22**, 12–18.

[8] Levy, J., Gavin, J. R., Hammerman, M. R. and Avioli, L. V. (1986). Ca/Mg ATPase activity in kidney basolateral membrane in non-insulin-dependent diabetic rats:Effects of insulin, *Diabetes*, **35**, 899–905.

[9] Draznin, B., Lewis, D., Houlder, N., Sherman, N., Adamo, M., Garvey, W. T., LeRoith, D. and Sussman, K. (1989). Mechanism of insulin resistance induced by sustained levels of cytosolic free calcium in rat adipocytes, *Endocrinology*, **125**, 2341–2349.

[10] Takaya, J., Iwamoto, Y., Higashino, H., Ishihara, R. and Kobayashi, Y. (1997). Increased intracellular calcium and altered phorbol dibutyrate binding to intact platelets in young subjects with insulin-dependent and non-insulin-dependent diabetes mellitus, *Metabolism*, **46**, 949–953.

[11] IeP, K. (1998). Changes in calcium homeostasis in the development of diabetes mellitus, *Fiziol Zh.*, **44**, 15–31.

[12] Rosskopf, D., Fromter, E. and Siffert, W. (1993). Hypertensive sodium-proton exchanger phenotype persists in immortalized lymphoblasts from essential hypertensive patients: A cell culture model for human hypertension, *J. Clin. Invest.*, **92**, 2553–2559.

[13] Balasubramanyam, M., Kimura, M., Aviv, A. and Gardner, J. P. (1993). Kinetics of $Ca^{2+}$ transport across the lymphocyte plasma membrane, *Am. J. Physiol.*, **265**, C321–327.

[14] Balasubramanyam, M., Rohowsky-Kochan, C., Reeves, J. P. and Gardner, J. P. (1994). Na/Ca exchange mediated calcium entry in human lymphocytes, *J. Clin. Invest.*, **94**, 2002–2008.

[15] World Health Organization (WHO). (1985). Diabetes Mellitus: Report of a WHO study group, Geneva, *World Health Org., Tech. Rep. Ser.*, **727**.

[16] Boyum, A. (1984). Separation of lymphocytes, granulocytes and monocytes from human blood using iodinated density gradient media, *Meth. Enzymol.*, **108**, 88–102.

[17] Scharschmidt, B. F., Keefee, E. B., Blankenship, N. M. and Ockner, R. K. (1979). Validation of a recording spectrophotometric method for measurement of membrane associated ATPase activity, *J. Lab. Clin. Med.*, **93**, 790–799.

[18] Grynkiewicz, G., Poenie, M. and Tsien, R. Y. (1985). A new generation of $Ca^{2+}$ indicators with greatly improved fluorescence properties, *J. Biol. Chem.*, **260**, 3440–3450.

[19] Lytton, J., Westlin, M. and Hanley, M. R. (1991). Thapsigargin inhibits the sarcoplasmic or endoplasmic reticulum $Ca^{2+}$-ATPase family of $Ca^{2+}$ pumps, *J. Biol. Chem.*, **266**, 17067–17071.

[20] Quinton, T. M. and Dean, W. L. (1992). Cyclic AMP-dependent phosphorylation of the inositol-1,4,5-triphosphate receptor inhibits $Ca^{2+}$ release from platelet membranes, *BBRC*, **184**, 893–899.

[21] Bruschi, G., Bruschci, M. E., Coroppo, M., Orlandini, G., Spaggiari, M. and Cavatorta, A. (1985). Cytoplasmic free Ca is increased in the platelets of spontaneously hypertensive rats and essential hypertensive patients, *Clin. Sci.*, **68**, 179–184.

[22] Mazzanti, L., Rabini, R. A., Faloia, E., Fumelli, P., Bertoli, E. and DePirro, R. (1990). Altered cellular $Ca^{2+}$ and $Na^{+}$ transport in diabetes mellitus, *Diabetes*, **39**, 850–854.

[23] Ishii, H., Umeda, F., Hashimoto, T. and Nawata, H. (1991). Increased intracellular calcium mobilization in platelets from patients with Type 2 (non-insulin-dependent) diabetes mellitus, *Diabetologia*, **34**, 332–336.

[24] Tschole, D., Rosen, P. and Gries, F. A. (1991). Increase in the cytosolic concentration of calcium in platelets of diabetes Type II, *Thromb Res.*, **62**, 421–428.

[25] Zentay, Z., Raguwanshi, M., Reddi, A., Lasker, N., Dasmahapatra, A. and Aviv, A. (1995). Cytosolic Ca profile of resting and thrombin-stimulated platelets from black women with NIDDM, *J. Diab. Complic.*, **9**, 74–80.

[26] Rivera, A., Conlin, P. R., Williams, G. H. and Canessa, M. L. (1996). Elevated lymphocyte cytosolic calcium in a subgroup of essential hypertensive subjects, *Hypertension*, **28**, 213–218.

[27] Balasubramanyam, M. and Gardner, J. P. (1997). Lymphocyte calcium homeostasis: a cellular model for studying physiology and pathophysiology, *Physiological Society Dublin (Ireland) meeting Abstracts*, p. 178.

[28] Watala, C., Boncer, M., Golanski, J., Koziolkiewcz, W., Trojanowski, Z. and Walkowiak, B. (1998). Platelet membrane lipid fluidity and intraplatelet calcium mobilization in Type 2 diabetes mellitus, *Eur. J. Haematol.*, **61**, 319–326.

[29] Erne, P., Bolli, P., Burgisser, E. and Buhler, F. R. (1984). Correlation of platelet calcium with blood pressure: effect of antihypertensive therapy, *New Engl. J. Med.*, **310**, 1084–1088.

[30] Litchtman, A. H., Sege, G. B. and Lichtman, M. A. (1981). Calcium transport and calcium-ATPase activity in human lymphocyte plasma membrane vesicles, *J. Biol. Chem.*, **256**, 6148–6154.

[31] Agarwal, V. R., Rastogi, A. K., Sahib, M. K. and Sagar, P. (1985). *In vitro* insulin action on different ATPases of erythrocyte membranes in normal and diabetic rats, *Acta Diabetol Lat.*, **22**, 111–118.

[32] Schaefer, W., Priessen, J., Mannhold, R. and Gries, A. F. (1987). $Ca^{2+}$-$Mg^{2+}$-ATPase activity of human red blood cells in healthy and diabetic volunteers, *Klin Wschr.*, **65**, 17–21.

[33] Makino, N., Dhalla, K. S., Elimban, V. and Dhalla, N. S. (1987). Sarcolemmal $Ca^{2+}$ transport in streptozotocin-induced diabetic cardiomyopathy in rats, *Am. J. Physiol.*, **253**, E202–E207.

[34] Allo, S. N., Lincoln, T. M., Wilson, G. L., Green, F. J., Watanabe, A. M. and Schaffer, S. W. (1991). Non-insulin-dependent diabetes induced defects in cardiac cellular calcium regulation, *Am. J. Physiol.*, **260**, C1165–C1171.

[35] Reddi, A. S., Dasmahapatra, A., Jyothirmayi, G. N. and Jayasundaramma, B. (1992). Erythrocyte Ca, Na/K-ATPase in long-term streptozotocin diabetic rats: effect of good glycemic control and a Ca antagonist, *Am. J. Hypertens.*, **5**, 863–868.

[36] Spieker, C., Fischer, S., Zierden, E., Schluter, H., Tepel, M. and Zidek, W. (1994). Cellular $Ca^{2+}$ ATPase activity in diabetes mellitus, *Horm Metab. Res.*, **26**, 544–547.

[37] Fu, Y., Wang, S. and Lu, Z. (1997). *The relationship between insulin resistance and abnormalities of cellular calcium metabolism and cell membrane abnormalities in patients with essential hypertension*, Chung Hua I Hsueh Tsa Chih., **77**, 443–446.

[38] Hoskins, B. and Scott, J. M. (1983). Calmodulin levels and divalent cation pump activities in kidneys of streptozotocin-diabetic rats, *Res. Commun. Chem. Pathol. Pharmacol.*, **39**, 189–199.

[39] Sahai, A. and Ganguly, P. K. (1991). ($Ca^{2+}$-$Mg^{2+}$) ATPase activity in kidney basolateral membrane in diabetes: role of atrial natriuretic peptide, *Mol. Cell. Biochem.*, **105**, 15–20.

[40] Takaya, J., Lasker, N., Bamforth, R., Gutkin, M., Byrd, L. H. and Aviv, A. (1990). Kinetics of $Ca^{2+}$ ATPase activation in platelet membranes of essential hypertensives and normotensives, *Am. J. Physiol.*, **258**, C988–C994.

[41] Dean, W. L., Pope, J. E., Brier, M. E. and Aronoff, G. R. (1993). Platelet calcium transport in hypertension, *Hypertension*, **23**, 31–37.

[42] Carafoli, E. (1991). Calcium pump of the plasma membrane, *Physiol Rev.*, **71**, 129–153.

[43] Penniston, J. T. and Enyedi, A. (1998). Modulation of the plasma membrane $Ca^{2+}$ pump, *J. Membr. Biol.*, **165**, 101–109.

[44] Furspan, P. and Bohr, D. (1985). Lymphocyte abnormalities in three types of hypertension in the rat, *Hypertension*, **7**, 860–866.

[45] Blaustein, M. P. and Hamlyn, J. M. (1991). Pathogenesis of essential hypertension, *Hypertension*, **18**, 184–195.

[46] Gardner, J. P. and Balasubramanyam, M. (1996). Na–Ca exchange in circulating blood cells, *Annals of NY Acad. Sci.*, **779**, 502–514.

[47] Wasada, T., Kuroki, H., Naruse, M., Arii, H., Maruyama, A., Katsumori, K., Saito, S., Watanabe, Y., Naruse, K., Demura, H. and Omori, Y. (1995). Insulin resistance is associated with high plasma ouabain-like immunoreactivity concentration in NIDDM, *Diabetologia*, **38**, 792–797.

[48] Liu, Y. J. and Gylfe, E. (1997). Store-operated $Ca^{2+}$ entry in insulin-releasing pancreatic beta-cells, *Cell Calcium*, **22**, 277–286.

[49] Hardie, R. C. and Minke, B. (1993). Novel $Ca^{2+}$ channels underlying transduction in Drosophila photoreceptors: implications for phosphoinositide-mediated $Ca^{2+}$ mobilization, *Trends Neurosci.*, **16**, 371–376.

[50] Sakura, H. and Ashcroft, F. M. (1997). Identification of four trp1 gene variants in murine pancreatic beta-cells, *Diabetologia*, **40**, 528–532.

[51] Roe, M. W., Worley, J. F., Qian, F., Tamarina, N., Mittal, A. A., Dralyuk, F., Blair, N. T., Mertz, R. J., Phillipson, L. H. and Dukes, I. D. (1998). Characterization of a $Ca^{2+}$ release-activated nonselective cation current regulating membrane potential and $Ca_i$ oscillations in transgenically derived beta-cells, *J. Biol. Chem.*, **273**, 10402–10410.

[52] Groschner, K., Hingel, S., Lintschinger, B., Balzer, M., Romanin, C., Zhu, X. and Schreibmayer, W. (1999). Trp proteins from store-operated cation channels in human vascular endothelial cells, *FEBS Lett.*, **437**, 101–106.

[53] Fekete, Z., Kimura, M., Horiguchi, M., Gardner, J. P., Nash, F. and Aviv, A. (1996). Differences between store-dependent $Ca^{2+}$ fluxes in lymphocytes from African Americans and whites, *J. Hypertension*, **14**, 1293–1299.

[54] Berridge, M. J. (1995). Capacitative calcium entry, *Biochem. J.*, **312**, 1–11.

[55] Balasubramanyam, M. and Gardner, J. P. (1995). Protein kinase C modulates cytosolic free calcium by stimulating calcium pump activity in *T* lymphocytes, *Cell Calcium*, **18**, 526–541.

[56] Xia, P., Inoguchi, T., Kern, T. S., Engerman, R. L., Oates, P. J. and King, G. L. (1994). Characterization of the mechanisms for the chronic activation of diacylglycerol-protein kinase C pathway in diabetes and hypergalactosemia, *Diabetes*, **43**, 1122–1129.

[57] Ohara, T., Sussman, K. E. and Draznin, B. (1991). Effect of diabetes on cytosolic free $Ca^{2+}$ and Na–K ATPase in rat aorta, *Diabetes*, **40**, 1560–1563.

[58] Sowers, J. R. (1992). Insulin resistance, hyperinsulinemia, dyslipidemia, hypertension and accelerated atherosclerosis, *J. Clin. Pharmacol.*, **32**, 529–535.

[59] Draznin, B. (1993). Cytosolic calcium and insulin resistance, *Am. J. Kidney Dis.*, **21**, 32–38.

[60] Fleischhacker, E., Esenabhalu, V. E., Spitaler, M., Holzmann, S., Skrabal, F., Koidl, B., Kostner, G. M. and Graier, W. F. (1999). Human diabetes is associated with hyperreactivity of vascular smooth muscle cells due to altered subcellular $Ca^{2+}$ distribution, *Diabetes*, **48**, 1323–1330.

[61] Levy, J. (1999). Abnormal cell calcium homeostasis in Type 2 diabetes mellitus: a new look on old disease, *Endocrine*, **10**, 1–6.

[62] Wehling, M. and Theisen, K. (1987). Altered calcium metabolism in red blood cells of hypertensives: persistent marker or sequel of essential hypertension?, *Klin Wochenschr.*, **65**, 769–772.

[63] Ng, L. L., Davies, J. E., Siczkowski, M., Sweeney, F. P., Quinn, P. A., Krolewski, B. and Krolewski, A. S. (1994). Abnormal $Na^+/H^+$ antiporter phenotype and turnover of immortalized lymphoblasts from Type 1 diabetic patients with nephropathy, *J. Clin. Invest.*, **93**, 2750–2757.

[64] Brzustowicz, L. M., Gardner, J. P., Hopp, L., Jeanclos, E., Ott, J., Yang, X. Y., Fekete, Z. and Aviv, A. (1997). Linkage analysis using platelet-activating factor $Ca^{2+}$ response in transformed lymphoblasts, *Hypertension*, **29**, 158–164.

[65] Varadi, A., Lebel, L., Hshim, Y., Mehta, Z., Ashcroft, S. J. H. and Turner, R. (1999). Sequence variants of the sarco(endo)plasmic reticulum $Ca^{2+}$-transport ATPase 3 gene (SERCA3) in Caucasian Type II diabetic patients (UK Prospective Diabetes Study 48), *Diabetologia*, **42**, 1240–1243.

